# Nitrogen Fertilization and Saline Solution on the Physiology, Micromorphology and Biochemical Components of Blueberry

**DOI:** 10.3390/plants15213350

**Published:** 2026-10-29

**Authors:** Luis-Enrique Espinoza-Orozco, Marcelino Cabrera-De-La Fuente, Ana V. Charles-Rodríguez, Antonio Juárez-Maldonado, Fabiola Aureoles-Rodríguez, José-Gerardo Uresti-Porras, Adalberto Benavides-Mendoza, Rocío Maricela Peralta Manjarrez

**Affiliations:** 1 Departamento de Horticultura, Universidad Autónoma Agraria Antonio Narro, Calzada Antonio Narro 1923, Saltillo C.P. 25315, Coahuila, Mexico; l.enrique.espinoza.orozco@gmail.com (L.-E.E.-O.); cafum7@yahoo.com (M.C.-D.-L.F.); fabyaureoles@yahoo.com.mx (F.A.-R.); abenmen@gmail.com (A.B.-M.); 2 Departamento de Ciencia y Tecnología de Alimentos, Universidad Autónoma Agraria Antonio Narro, Calzada Antonio Narro 1923, Saltillo C.P. 25315, Coahuila, Mexico; anavero29@gmail.com; 3 Departamento de Botánica, Universidad Autónoma Agraria Antonio Narro, Calzada Antonio Narro 1923, Saltillo C.P. 25315, Coahuila, Mexico; juma841025@gmail.com; 4 Facultad de Agronomía, Universidad Autónoma de Nuevo León, Francisco I. Madero S/N, Ex Hacienda el Canadá, General Escobedo C.P. 66050, Nuevo Leon, Mexico; gera_up@hotmail.com; 5 SEIHTI, Departamento de Horticultura, Universidad Autónoma Agraria Antonio Narro, Calzada Antonio Narro 1923, Saltillo C.P. 25315, Coahuila, Mexico

**Keywords:** Biloxi, nitrate, ammonium, salinity, net photosynthetic rate, CO_2_ assimilation, stomata, sugar, vitamin C

## Abstract

Nitrogen formulation and saline irrigation may influence blueberry physiology, stomatal characteristics, and fruit composition. This study evaluated four NH_4_^+^–N_3_^−^–N formulations (100:0, 75:25, 0:100, and 25:75) under two salinity conditions (0 and 30 mM NaCl), plus an additional 50:50 control without NaCl, in ‘Biloxi’ blueberry grown in coconut fiber under protected cultivation. Salinity was applied through weekly irrigation with 30 mM NaCl. Leaf gas exchange was measured in three independent plants per treatment, and stomatal micromorphology was evaluated in four plants per treatment. Fruit total sugars and ascorbic acid were determined descriptively from a single composite sample per treatment. Significant formulation × salinity interactions were observed for net photosynthetic rate (*A*), stomatal conductance (*g_s_*), intercellular CO_2_ concentration (*C_i_*), and transpiration rate (*E*). Salinity reduced *A* under the 100:0, 0:100, and 25:75 formulations, whereas the 75:25 formulation showed an increase from 2.733 to 4.500 µmol CO_2_ m^−2^ s^−1^. Nitrogen formulation significantly affected stomatal index and density, while salinity and its interaction with formulation were not significant for the evaluated micromorphological traits. Total sugars and ascorbic acid exhibited descriptive variation among treatments, but biological treatment effects could not be established. These findings demonstrate formulation-dependent physiological responses to intermittent saline irrigation and highlight the importance of considering the complete nutrient composition when interpreting blueberry responses.

## 1. Introduction

Blueberry (*Vaccinium corymbosum* L.) is valued for its nutritional and biochemical characteristics, particularly its sugar composition and antioxidant-related compounds [1,2]. Protected cultivation in soilless substrates provides opportunities to manage nutrient supply and irrigation conditions; however, the physiological and biochemical responses of blueberry plants depend on the interaction between nutritional and environmental factors [3,4].

Nitrogen (N) is essential for blueberry growth and photosynthetic metabolism, and its inorganic forms can differentially influence plant responses. Although blueberry frequently exhibits a preference for ammonium (NH_4_^+^), the effects of nitrogen nutrition vary with nutrient concentration and cultivation conditions, as reviewed by Doyle et al. [4]. Cárdenas-Navarro et al. [5] reported greater photosynthetic activity and several growth responses in blueberry supplied with 6.0 mM N as NH_4_^+^. In contrast, Anwar et al. [6] identified favorable photosynthetic and growth responses with a 50:50 NH_4_^+^–NO_3_^−^–N ratio under soilless cultivation.

Nitrogen nutrition may also influence leaf epidermal development and stomatal characteristics. Yang et al. [7] observed differences in stomatal dimensions among blueberry seedlings supplied with different nitrogen forms, whereas Sathee and Jain [8] documented changes in stomatal anatomy associated with nitrogen nutrition in wheat. Benevenute et al. [9] further demonstrated variability in stomatal morphology among blueberry genotypes, highlighting the importance of distinguishing stomatal density from stomatal dimensions.

Salinity represents an additional challenge for blueberry production because it can disrupt plant water relations, ion homeostasis, and photosynthetic processes [10,11]. Saline conditions associated with ammonium sulfate fertilization have been linked to reduced blueberry growth [12], while Ma et al. [13] documented differences in physiological responses to chloride stress among blueberry cultivars. Chloride may also interact with nitrate uptake and utilization, as discussed by Carillo and Rouphael [14] for vegetable crops.

Nitrogen management and saline conditions may influence fruit biochemical composition. Okan et al. [1] characterized variation in sugar composition among blueberry cultivars, while Duan et al. [15] reported changes in sugars and vitamin C associated with nitrogen forms in blackberry fruit. In ‘Biloxi’ blueberry, Bolaños-Alcántara et al. [16] examined nitrogen ratios and electrical conductivity in relation to fruit quality.

A previous study using the same experimental system evaluated the effects of nitrogen formulations and salinity on vegetative growth, flowering, yield, and physical fruit-quality traits in ‘Biloxi’ blueberry [17]. However, leaf gas exchange, stomatal micromorphology, and fruit total sugar and ascorbic acid contents were not reported in that publication. The present study addresses these complementary response variables to provide a more comprehensive characterization of blueberry responses to nutrient formulation and intermittent saline irrigation.

Therefore, this study aimed to evaluate the effects of four nominal NH_4_^+^–NO_3_^−^–N formulations (100:0, 75:25, 0:100, and 25:75) and intermittent irrigation with 0 or 30 mM NaCl on leaf gas exchange and stomatal micromorphology of ‘Biloxi’ blueberry grown in coconut fiber under protected cultivation. An additional 50:50 formulation without NaCl was included as a control. Total sugar and ascorbic acid contents in fruit were also characterized descriptively. We hypothesized that physiological and micromorphological responses would differ among nutrient formulations and their combinations with saline irrigation.

## 2. Results

Physiological Parameters The factorial analysis of variance revealed significant interactions between nitrogen formulation (F) and NaCl salinity (S) for net photosynthetic rate (*A*; F = 54.65, *p* < 0.0001), stomatal conductance (*g_s_*; F = 3.45, *p* = 0.0417), intercellular CO_2_ concentration (*C_i_*; F = 4.36, *p* = 0.0200), and transpiration rate (*E*; F = 5.80, *p* = 0.0070). Nitrogen formulation significantly affected *A* (*p* < 0.0001), whereas salinity showed significant main effects on *A* (*p* < 0.0001), *C_i_* (*p* = 0.0075), and *E* (*p* = 0.0497). Given the significant interactions, the physiological responses were primarily interpreted through comparisons among nitrogen formulations within each salinity level and between salinity conditions within each formulation (Table 1).

**Figure 1:**
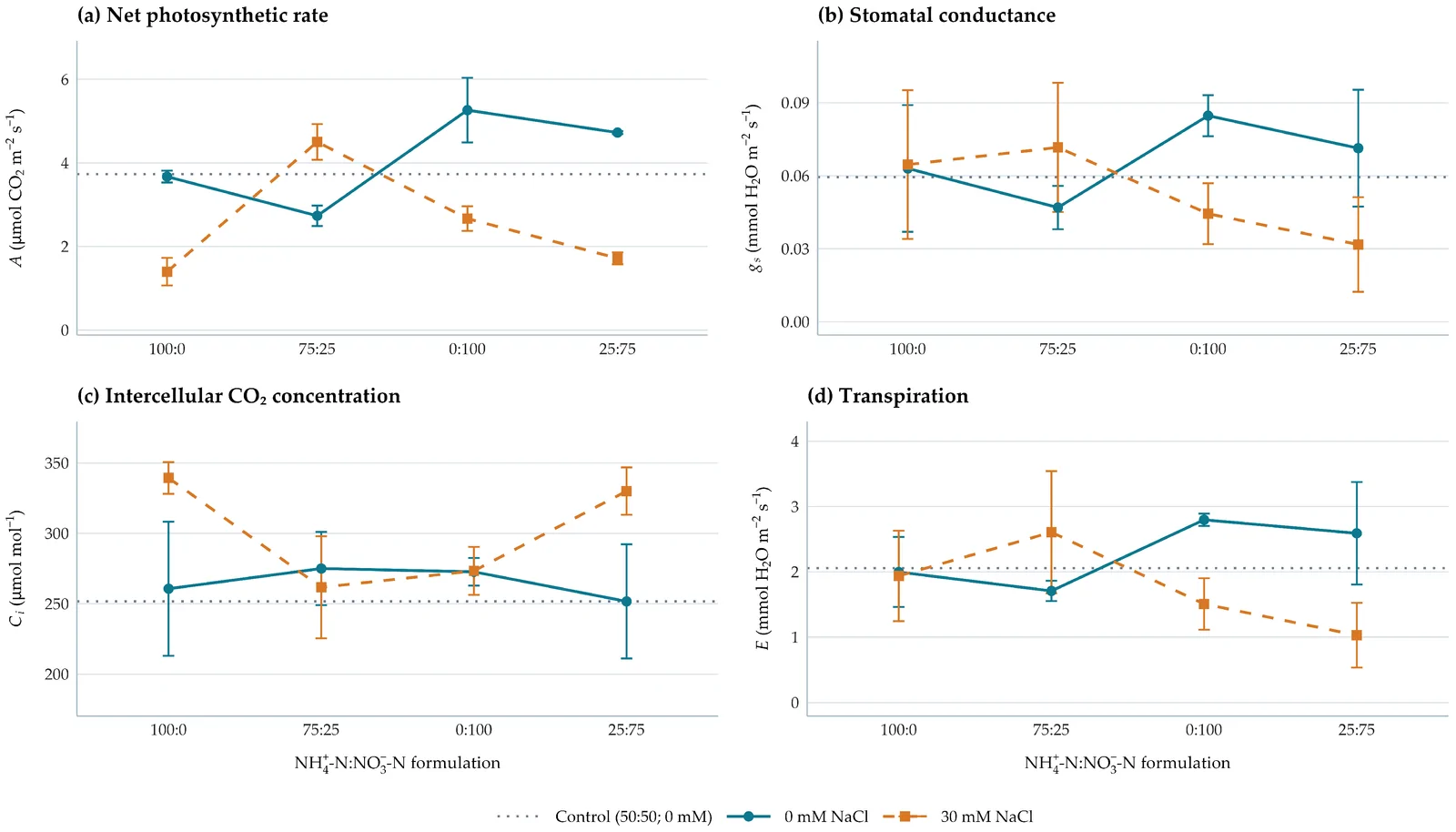
Interaction between nitrogen formulation and NaCl salinity on blueberry leaf gas exchange. Symbols represent treatment means and error bars show standard deviations of three independent plants (n = 3). The dotted horizontal line denotes the 50:50 NH_4_^+^–N:NO_3_^−^–N control without NaCl.

Net photosynthetic rate (*A*) differed considerably among nitrogen formulations and salinity conditions. In the absence of added NaCl, plants receiving the 0:100 and 25:75 NH_4_^+^–N_3_^−^–N formulations exhibited the highest photosynthetic rates, with mean values of 5.260 and 4.723 µmol CO_2_ m^−2^ s^−1^, respectively, without significant differences between these treatments. In contrast, the 100:0 and 75:25 formulations showed lower values of 3.670 and 2.733 µmol CO_2_ m^−2^ s^−1^, respectively. Under 30 mM NaCl, the response pattern changed markedly. The 75:25 formulation exhibited the highest photosynthetic rate (4.500 µmol CO_2_ m^−2^ s^−1^), significantly exceeding the other formulations, whereas the 100:0 and 25:75 formulations presented the lowest values (1.397 and 1.717 µmol CO_2_ m^−2^ s^−1^, respectively). The 0:100 formulation showed an intermediate response (2.667 µmol CO_2_ m^−2^ s^−1^) (Table 1; Figure 1a). Comparisons of salinity effects within each nitrogen formulation confirmed significant but contrasting photosynthetic responses. Relative to their corresponding non-saline treatments, the application of 30 mM NaCl reduced *A* by approximately 61.9%, 49.3%, and 63.7% under the 100:0, 0:100, and 25:75 formulations, respectively. Conversely, the 75:25 formulation showed a significant increase of approximately 64.6%, from 2.733 to 4.500 µmol CO_2_ m^−2^ s^−1^. All four comparisons remained significant after Holm adjustment (*p* < 0.05). These contrasting responses explain the significant interaction between nitrogen formulation and salinity for net photosynthesis (Table 1; Figure 1a). Stomatal conductance (*g_s_*) also exhibited a significant formulation × salinity interaction (*p* = 0.0417), although neither factor showed a significant main effect. Without added NaCl, the highest numerical value was observed under the 0:100 formulation (0.0847 mmol H_2_O m^−2^ s^−1^), whereas the lowest occurred under the 75:25 formulation (0.0469 mmol H_2_O m^−2^ s^−1^). Under salinity, the 75:25 formulation exhibited the highest numerical conductance (0.0717 mmol H_2_O m^−2^ s^−1^), while the 25:75 formulation showed the lowest value (0.0317 mmol H_2_O m^−2^ s^−1^). Nevertheless, Tukey comparisons did not identify significant differences among formulations within either salinity condition. Similarly, none of the individual salinity contrasts remained significant after Holm adjustment (*p* > 0.05). Therefore, despite the significant overall interaction, the observed differences in *g_s_* among individual treatment combinations should be interpreted cautiously (Table 1; Figure 1b). Intercellular CO_2_ concentration (*C_i_*) was significantly affected by salinity (*p* = 0.0075) and by its interaction with nitrogen formulation (*p* = 0.0200), whereas the main effect of formulation was not significant. Under non-saline conditions, *C_i_* ranged from 251.7 to 275.0 µmol CO_2_ mol^−1^, without significant differences among formulations. Following the application of 30 mM NaCl, the 100:0 and 25:75 formulations exhibited the highest numerical values (339.3 and 330.0 µmol CO_2_ mol^−1^, respectively), while the 75:25 formulation showed the lowest value (261.7 µmol CO_2_ mol^−1^). Within-formulation comparisons indicated that salinity significantly increased *C_i_* by 78.7 and 78.3 µmol CO_2_ mol^−1^ under the 100:0 and 25:75 formulations, respectively (Holm-adjusted *p* < 0.05). No significant salinity-induced changes were detected in the 75:25 or 0:100 formulations. These results indicate that the effect of saline irrigation on intercellular CO_2_ concentration differed among the evaluated nutrient formulations (Table 1; Figure 1c).

Transpiration rate (*E*) showed significant effects of salinity (*p* = 0.0497) and the formulation × salinity interaction (*p* = 0.0070), while the main effect of nitrogen formulation was not significant. In the absence of added NaCl, *E* ranged from 1.707 to 2.797 mmol H_2_O m^−2^ s^−1^, with no significant differences among the four formulations. Under 30 mM NaCl, the 75:25 formulation maintained the highest numerical transpiration rate (2.607 mmol H_2_O m^−2^ s^−1^), whereas the 25:75 formulation exhibited the lowest value (1.028 mmol H_2_O m^−2^ s^−1^); these two treatments differed significantly according to Tukey’s test. Comparisons within formulations showed that salinity significantly reduced *E* under the 0:100 formulation, from 2.797 to 1.507 mmol H_2_O m^−2^ s^−1^, and under the 25:75 formulation, from 2.590 to 1.028 mmol H_2_O m^−2^ s^−1^ (Holm-adjusted *p* < 0.05). No significant salinity effects were detected for *E* in the 100:0 or 75:25 formulations (Table 1; Figure 1d).

The additional 50:50 NH_4_^+^–N_3_^−^–N control treatment, evaluated without added NaCl, showed mean values of 3.730 µmol CO_2_ m^−2^ s^−1^ for *A*, 0.0594 mmol H_2_O m^−2^ s^−1^ for *g_s_*, 251.7 µmol CO_2_ mol^−1^ for *C_i_*, and 2.057 mmol H_2_O m^−2^ s^−1^ for *E*. Dunnett-adjusted comparisons indicated that photosynthesis was significantly higher than the control under T5 and T7 and significantly lower under T2, T3, T6, and T8 (*p* < 0.05). Treatments T1 and T4 did not differ significantly from the control in *A*. For *C_i_*, only T2 showed a significant difference relative to the control, whereas no significant differences were detected for *g_s_* or *E*. These control comparisons were performed separately from the factorial analysis (Table 1).

Overall, gas-exchange responses differed according to the specific combination of nitrogen formulation and salinity. The contrasting photosynthetic responses were particularly evident under the 75:25 formulation, which exhibited increased *A* under saline irrigation, unlike the reductions observed in the other three formulations. Although stomatal conductance, intercellular CO_2_ concentration, and transpiration also exhibited significant formulation × salinity interactions, their responses were variable, and not all individual treatment comparisons reached statistical significance. Together, these findings emphasize the importance of evaluating individual nutrient formulations and their responses to salinity rather than relying exclusively on the main effects of nitrogen form or salinity.

### 2.1. Stomatal Micromorphological Results

The factorial analysis of variance revealed significant effects of nitrogen formulation on stomatal index (*SI*; F = 12.33, *p* < 0.0001) and stomatal density (*SD*; F = 8.37, *p* = 0.00055). Neither salinity nor the formulation × salinity interaction significantly affected these variables (*p* > 0.05). Stomatal length (*SL*), width (*SW*), area (*SA*), and perimeter (*SP*) were not significantly affected by nitrogen formulation, salinity, or their interaction (Table 2).

**Figure 2:**
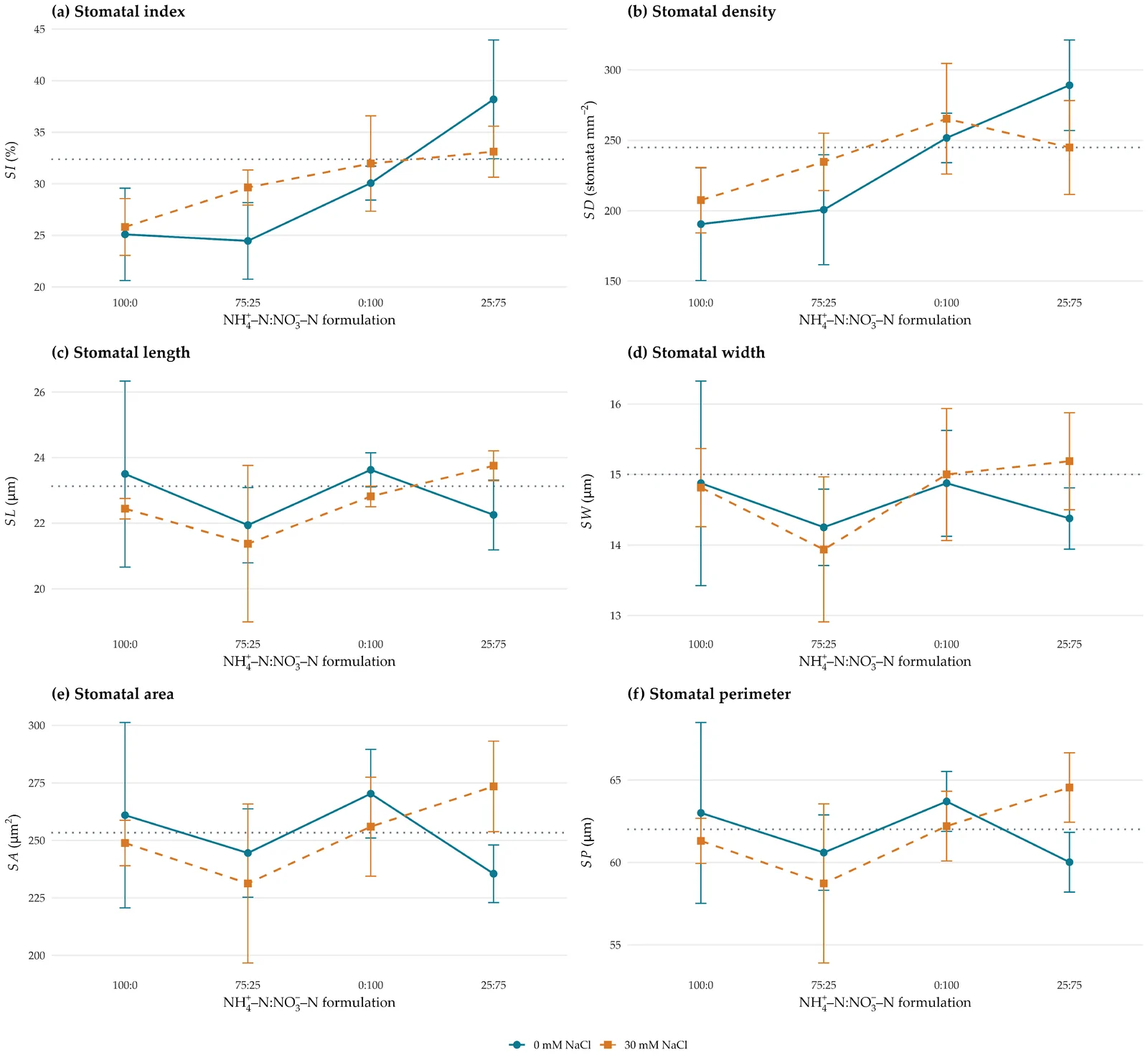
Effects of nitrogen formulation and NaCl salinity on leaf stomatal micromorphological traits of blueberry cv. ‘Biloxi’: (**a**) stomatal index (*SI*), (**b**) stomatal density (*SD*), (**c**) stomatal length (*SL*), (**d**) stomatal width (*SW*), (**e**) stomatal area (*SA*), and (**f**) stomatal perimeter (*SP*). Symbols represent treatment means, and error bars indicate standard deviations of four independent plants per treatment (n = 4). Dotted horizontal lines indicate the corresponding mean values of the additional 50:50 NH_4_^+^–NO_3_^−^–N control without added NaCl.

Under non-saline conditions, the 25:75 NH_4_^+^–NO_3_^−^–N formulation exhibited the highest *SI* (38.19%), significantly exceeding the 100:0 (25.09%), 75:25 (24.46%), and 0:100 (30.06%) formulations. Under 30 mM NaCl, the 25:75 formulation also showed the highest numerical *SI* (33.11%), although significant differences were detected only relative to the 100:0 formulation (25.81%). The 75:25 and 0:100 formulations presented intermediate values (Table 2; Figure 2a).

Stomatal density (*SD*) showed a similar pattern. Without added NaCl, the 25:75 formulation exhibited the highest *SD* (289.11 stomata mm^−2^), significantly exceeding the 100:0 and 75:25 formulations (190.48 and 200.68 stomata mm^−2^, respectively). The 0:100 formulation showed an intermediate value (251.70 stomata mm^−2^). Under saline conditions, *SD* ranged from 207.48 to 265.30 stomata mm^−2^, without significant differences among formulations (Table 2; Figure 2b).

Although numerical differences in *SI* and *SD* were observed between salinity conditions, neither variable showed a significant main effect of salinity. Averaged across the four nitrogen formulations, *SI* was 29.45% at 0 mM and 30.13% at 30 mM NaCl, whereas *SD* was 232.99 and 238.09 stomata mm^−2^, respectively. These results indicate that no statistically significant overall changes in stomatal index or density were detected following saline irrigation (Table 2).

Stomatal dimensions exhibited relatively limited numerical variation among treatments. Across the nine treatments, *SL* ranged from 21.38 to 23.75 µm, *SW* from 13.94 to 15.19 µm, *SA* from 231.27 to 273.46 µm^2^, and *SP* from 58.73 to 64.55 µm. No significant differences among formulations were detected within either salinity level, consistent with the non-significant factorial effects for these traits (Table 2; Figure 2c–f). These measurements corresponded to the entire paired guard-cell structure rather than exclusively to the central stomatal pore.

The additional 50:50 NH_4_^+^–NO_3_^−^–N control treatment presented an *SI* of 32.38% and an *SD* of 244.90 stomata mm^−2^. Dunnett-adjusted comparisons identified a significantly lower *SI* in T3 (75:25 without NaCl) than in the control (*p* < 0.05). No other significant differences relative to the control were detected for *SI*, *SD*, *SL*, *SW*, *SA*, or *SP* (Table 2).

Overall, nitrogen formulation significantly affected stomatal index and density, whereas no significant effects of salinity or formulation × salinity interactions were detected. Stomatal dimensions remained statistically comparable among the evaluated treatment combinations, indicating that the observed micromorphological differences were primarily associated with stomatal frequency rather than changes in guard-cell dimensions.

### 2.2. Descriptive Fruit Biochemical Results

The biochemical analysis revealed numerical differences in total sugars (TS) and ascorbic acid (AA) among nitrogen formulations and salinity conditions (Table 3). These results were evaluated descriptively because fruits from the ten plants within each treatment were pooled into a single composite sample. Therefore, the reported means and standard deviations represent three independent analytical preparations rather than biological replication.

**Figure 3:**
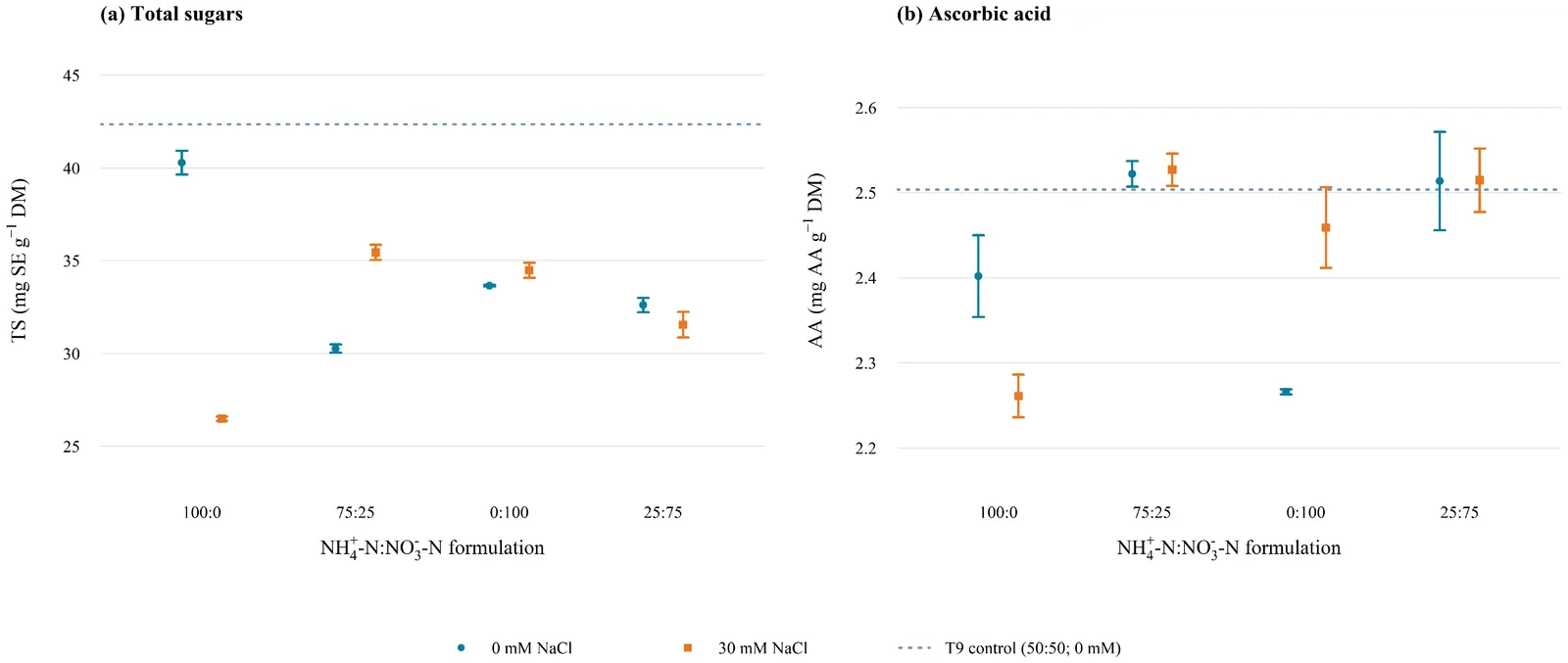
Descriptive variation in fruit biochemical composition of blueberry cv. ‘Biloxi’ under different nitrogen formulations and NaCl salinity conditions: (**a**) total sugars (TS, mg SE g^−1^ DM) and (**b**) ascorbic acid (AA, mg AA g^−1^ DM). Symbols represent means of three independently extracted analytical subsamples from a single composite fruit sample per treatment, and error bars indicate analytical standard deviations. Dotted horizontal lines represent the corresponding mean values of the additional 50:50 NH_4_^+^–NO_3_^−^–N control without added NaCl. SE, sucrose equivalents; AA, ascorbic acid; DM, dry matter.

Total sugar content (TS) ranged from 26.49 to 40.28 mg SE g^−1^ DM among the eight factorial treatments. The highest numerical value was recorded under the 100:0 NH_4_^+^–NO_3_^−^–N formulation without NaCl (40.28 mg SE g^−1^ DM), whereas the lowest corresponded to the same formulation under 30 mM NaCl (26.49 mg SE g^−1^ DM). This represented a descriptive reduction of approximately 34.2% (Table 3; Figure 3a).

The response of TS to saline irrigation varied among formulations. Under the 75:25 formulation, TS increased numerically from 30.27 to 35.45 mg SE g^−1^ DM, whereas the 0:100 and 25:75 formulations exhibited smaller differences between salinity conditions (33.66–34.49 and 32.62–31.55 mg SE g^−1^ DM, respectively). The additional 50:50 control presented the highest numerical TS content (42.35 mg SE g^−1^ DM) among all nine treatments (Table 3; Figure 3a).

Ascorbic acid content (AA) showed a relatively narrow range, from 2.261 to 2.527 mg AA g^−1^ DM. Under the 100:0 formulation, the numerical value decreased from 2.402 to 2.261 mg AA g^−1^ DM with saline irrigation. In contrast, the 0:100 formulation exhibited an increase from 2.266 to 2.459 mg AA g^−1^ DM. The 75:25 and 25:75 formulations showed nearly unchanged values between salinity conditions (Table 3; Figure 3b).

The 50:50 control presented an AA content of 2.504 mg AA g^−1^ DM, comparable numerically to those observed under the 75:25 and 25:75 formulations. Overall, the descriptive patterns indicated that TS exhibited greater variation among treatment combinations than AA. However, because biological replication was unavailable for these assays, the observed numerical differences cannot be interpreted as statistically established effects of nitrogen formulation, salinity, or their interaction.

## 3. Discussion

Physiological Responses to Nitrogen Formulation and NaCl SalinityThe significant formulation × salinity interactions observed for A, g_s_, C_i_, and E indicate that gas-exchange responses in ‘Biloxi’ blueberry depended on the specific nutrient formulation. Blueberry nitrogen acquisition and assimilation are influenced by the supplied inorganic N form and its interaction with plant nutritional requirements, as reviewed by Doyle et al. [4], nitrogen acquisition and assimilation in blueberry are closely associated with plant nutritional requirements. Britto and Kronzucker [18] further emphasized that plant preference for inorganic N forms depends on interacting physiological and environmental factors.

Under non-saline conditions, the 0:100 and 25:75 NH_4_^+^–NO_3_^−^–N formulations showed the highest net photosynthetic rates. However, this response does not indicate a general superiority of nitrate. Cárdenas-Navarro et al. [5] reported greater photosynthesis in blueberry supplied with 6.0 mM N as NH_4_^+^, whereas Anwar et al. [6] identified a favorable photosynthetic response with a 50:50 NH_4_^+^–N_3_^−^–N ratio. These differences suggest that responses depend on the nutritional formulation and experimental conditions.

The present physiological findings complement the agronomic responses previously reported from the same experiment. Espinoza-Orozco et al. [17] found greater biomass, flowering, fruit production, and yield under ammonium-dominated treatments. In contrast, nitrate-dominated formulations exhibited higher instantaneous net photosynthetic rates under non-saline conditions in the present analysis. These findings are not necessarily contradictory, as leaf gas exchange measured at a single developmental stage does not directly represent cumulative carbon assimilation, biomass allocation, or seasonal yield.

Differences in nitrogen assimilation may contribute to these responses. Nitrate reduction involves nitrate reductase activity [19] and is closely integrated with carbon metabolism [20,21,22]. In contrast, NH_4_^+^ assimilation occurs mainly through the GS/GOGAT pathway [23], while its uptake and distribution involve regulated ammonium transport systems [24]. However, excessive ammonium supply may produce physiological and nutritional imbalances [25,26]. These processes provide possible explanations for treatment differences but were not directly evaluated in this study.

Saline irrigation significantly reduced *A* under the 100:0, 0:100, and 25:75 formulations, whereas the 75:25 formulation showed an opposite response. Salgado Vargas et al. [27] documented adverse effects of osmotic stress on blueberry performance, while Ma et al. [13] reported cultivar-dependent physiological responses to chloride stress. Salinity associated with ammonium sulfate fertilization has also been linked to reduced blueberry growth [12]. These findings support the sensitivity of blueberry to saline conditions, although they do not explain the increased photosynthesis observed under the 75:25 formulation.

The simultaneous reduction in *A* and increase in *C_i_* under the 100:0 and 25:75 formulations suggest that photosynthetic limitations may not have been exclusively stomatal. Cao et al. [28] documented chloroplast damage under prolonged salt exposure in Jatropha curcas, while Wang et al. [24] described chloroplast responses to salinity. Similarly, Lu et al. [29] reported reductions in photosynthesis associated with chloroplast structural alterations in Robinia pseudoacacia. However, these mechanisms cannot be confirmed in the present study because chlorophyll fluorescence and chloroplast characteristics were not measured.

Although the formulation × salinity interaction was significant for *g_s_*, individual comparisons did not reveal significant differences after adjustment for multiple comparisons. Therefore, the numerical changes should be interpreted cautiously. Gupta and Huang [10] described the complexity of physiological responses to salinity, while Balasubramaniam et al. [30] emphasized the involvement of water relations and cellular signaling in plant stress responses.

Salinity increased *C_i_* while reducing *A* under the 100:0 and 25:75 formulations. This pattern suggests that the decrease in CO_2_ assimilation may not be explained exclusively by stomatal limitations. Salinity-induced alterations in chloroplast structure and function have been documented in *Jatropha curcas* [28], while Wang et al. [24] reviewed the sensitivity of chloroplasts to salt stress. However, chlorophyll fluorescence and photosynthetic biochemical capacity were not measured in our study; thus, non-stomatal limitations remain a possible explanation.

Transpiration rate (*E*) decreased significantly under salinity in the 0:100 and 25:75 formulations. Lu and Fricke [23] described how salt stress can affect root water uptake and hydraulic regulation. Likewise, Van Zelm et al. [31] emphasized the importance of ion homeostasis and osmotic adjustment in plant salt tolerance. Although these processes could contribute to the observed reductions in *E*, plant water potential and hydraulic conductivity were not evaluated.

The physiological responses must also be interpreted considering differences in the accompanying nutrients. The formulations varied in potassium, calcium, and sulfur concentrations, which may have contributed to the observed treatment effects. Doyle et al. [4] emphasized the importance of mineral nutrition in blueberry physiology, while Carillo and Rouphael [14] described interactions between chloride and nitrate uptake in vegetable crops. Consequently, the observed responses cannot be attributed exclusively to the NH_4_^+^ NO_3_^−^ ratio.

Finally, salinity was imposed through weekly irrigation with 30 mM NaCl, replacing the nutrient solution on those days. Because substrate salinity was not monitored and treatment positions were not randomly assigned, the results should be interpreted within these experimental limitations. Overall, the findings emphasize that physiological performance depended on the specific nutrient formulation and saline irrigation regime, rather than demonstrating a universal advantage of either nitrogen form.

### 3.1. Stomatal Micromorphological Responses to Nitrogen Formulation and NaCl Salinity

Nitrogen formulation significantly affected stomatal index (*SI*) and stomatal density (*SD*) in ‘Biloxi’ blueberry, with the highest values observed under the 25:75 NH_4_^+^–NO_3_^−^–N formulation without added NaCl. Sathee and Jain [8] demonstrated that nitrogen form can influence leaf epidermal and stomatal anatomy in wheat seedlings. Although their experimental conditions differed, these findings support the potential relationship between nitrogen nutrition and stomatal characteristics.

Differences in stomatal characteristics have also been reported in blueberry. Yang et al. [7] observed greater stomatal length and width in seedlings supplied with NH_4_^+^ than with NO_3_^−^. In contrast, the present study did not detect significant effects of nitrogen formulation on stomatal length (*SL*), width (*SW*), area (*SA*), or perimeter (*SP*). These differences may be related to cultivar, developmental stage, and nutrient formulation.

Benevenute et al. [9] documented considerable variation in stomatal area and density among cultivated blueberry genotypes and examined their relationships with responses to irradiance. Although the present experiment evaluated a single cultivar, these findings highlight the importance of considering stomatal density and size as distinct anatomical traits.

Salinity did not significantly affect *SI*, *SD*, *SL*, *SW*, *SA*, or *SP*, and no significant formulation × salinity interactions were detected. Matkowski and Daszkowska-Golec [32] described the influence of abiotic stress on stomatal development and functioning. However, the absence of significant differences in the present study does not necessarily indicate complete anatomical stability or salinity tolerance, particularly because NaCl was applied intermittently and root-zone salinity was not monitored.

Stomatal characteristics can influence gas exchange through their effects on CO_2_ diffusion and water-vapor movement. Harrison et al. [33] emphasized that the relationship between stomatal morphology, density, and photosynthetic performance is complex. In the present study, significant differences in *SI* and *SD* were not consistently accompanied by corresponding differences in *g_s_* or *A*. Therefore, the observed micromorphological patterns cannot independently explain the physiological responses.

Furthermore, *SL*, *SW*, *SA*, and *SP* were measured across the external boundaries of the paired guard cells rather than exclusively within the stomatal pore. Consequently, these variables describe stomatal structure but do not directly quantify pore aperture or stomatal opening. The absence of significant differences in these dimensions should therefore not be interpreted as evidence of unchanged stomatal function.

Finally, differences in accompanying mineral nutrients, particularly K, Ca, and S, may have contributed to the observed anatomical responses. Thus, the effects cannot be attributed exclusively to the NH_4_^+^–NO_3_^−^–N ratio. Overall, the results indicate that nutrient formulation was associated with differences in stomatal index and density, whereas the evaluated salinity treatment did not produce statistically detectable changes in stomatal micromorphology.

### 3.2. Fruit Biochemical Composition Under Nitrogen Formulation and NaCl Salinity

Total sugar content (TS) showed numerical variation among nitrogen formulations and salinity conditions, with the highest value recorded in the 50:50 control. Okan et al. [1] documented differences in sugar composition among blueberry cultivars, highlighting the variability of this fruit characteristic. Similarly, Liu et al. [2] reported considerable differences in biochemical quality among highbush blueberry cultivars.

Under the 100:0 NH_4_^+^–NO_3_^−^–N formulation, saline irrigation was associated with a numerical reduction in TS from 40.28 to 26.49 mg SE g^−1^ DM. However, the remaining formulations showed different patterns. Bolaños-Alcántara et al. [16] evaluated nitrogen ratios and electrical conductivity in ‘Biloxi’ blueberry, although they found no significant differences in total soluble solids. Thus, their findings provide a relevant nutritional context but are not directly comparable with the total sugar measurements obtained in the present study.

Nitrogen nutrition may influence carbohydrate accumulation in berry crops. Duan et al. [15] reported that ammonium nutrition increased fruit sugar accumulation in blackberry, whereas Anwar et al. [6] observed changes in soluble sugars and starch in blueberry leaves under different NH_4_^+^–NO_3_^−^–N ratios. Nevertheless, differences in plant species, sampled tissues, and analytical methods prevent direct extrapolation of these findings to the present fruit samples.

Ascorbic acid (AA) exhibited less numerical variation than TS among the evaluated treatments. Duan et al. [15] demonstrated that nitrogen form influenced vitamin C accumulation in blackberry fruit, while Liu et al. [2] identified differences in vitamin C content among highbush blueberry cultivars. These studies indicate that ascorbic acid content may vary with nutritional and biological conditions, although the present results do not establish treatment-related effects.

The biochemical results must be interpreted considering the analytical methodology. Total sugars were determined using the phenol–sulfuric acid method [20,34], whereas AA was quantified using the DCPIP colorimetric procedure adapted from Manríquez-Torres et al. [35]. Therefore, the measurements represent sucrose-equivalent carbohydrates and ascorbic acid, respectively, rather than a detailed characterization of individual sugars or the complete antioxidant profile of the fruit.

Furthermore, fruits from all ten plants within each treatment and different harvest dates were combined into a single composite sample. Although three independent analytical preparations were performed, they did not constitute independent biological replicates. Consequently, the numerical differences in TS and AA cannot be considered statistically demonstrated effects of nitrogen formulation or salinity.

Overall, the descriptive results suggest variation in fruit biochemical composition among the evaluated nutrient formulations and saline irrigation conditions. However, further studies using independent biological samples, controlled nutrient compositions, and additional biochemical determinations are required to establish whether these treatments consistently modify sugar accumulation and ascorbic acid content in ‘Biloxi’ blueberry fruit.

## 4. Materials and Methods

The research was carried out under low-technology greenhouse conditions in La Ascensión, Aramberri, Nuevo León, México. This location is situated at 24°20′14.96″ N latitude and 99°56′9.5″ W longitude, at an altitude of 1961 m above sea level, where the average annual temperature is 15.6 °C. The greenhouse was covered with a 180-µm-thick (720-gauge) white polyethylene film (Suntherm White 25%, Ginegar Plastic Products Ltd., Kibbutz Ginegar, Israel), with a nominal shading level of 25%, and was equipped with anti-aphid mesh. During the experimental period, the mean maximum and minimum air temperatures recorded inside the greenhouse were 21.3 and 12.6 °C, respectively. Relative humidity and incident solar radiation inside the greenhouse were not monitored.

### 4.1. Plant Material

The plants used were blueberries (*Vaccinium corymbosum* L.) of the variety ‘Biloxi’, obtained from an in vitro propagation system. They were acquired in 1-L pots on May 22, 2023. During the adaptation period, the plants received a fertilization solution based on the following concentrations (g L^−1^): 0.1101 K_2_SO_4_, 0.0440 KH_2_PO_4_, 0.2150 Ca(NO_3_)_2_·4H_2_O, 0.1070 MgSO_4_·7H_2_O, 0.1350 (NH_4_)_2_SO_4_, and 0.02 of a micronutrient mix. This provided a contribution in ppm of: Fe 1.5, Mn 0.74, B 0.08, Zn 0.12, Cu 0.06, and Mo 0.04. The solution was prepared with distilled water, and its composition was based on the results reported by Frías-Ortega et al. [3].

### 4.2. Plant Transplantation

Transplanting took place on 2 October 2023, using black plastic growing bags measuring 0.30 m in diameter and 0.37 m in height, each filled with 20 L of pre-washed, medium-textured coconut fiber as the substrate. This material was selected for its ability to retain moisture and provide adequate gas exchange for the root system. The plants were carefully removed from their 1 L containers to minimize root damage. They were then placed on the coconut fiber, ensuring direct contact between the roots and the substrate to optimize water and nutrient absorption. Finally, initial watering was applied to promote plant establishment, using the appropriate nutrient solution for the acclimation period.

### 4.3. Experimental Design and Treatments

The experiment comprised nine treatment regimes, of which eight formed a 4 × 2 factorial structure consisting of four nominal NH_4_^+^–NO_3_^−^–N formulations (100:0, 75:25, 0:100, and 25:75) and two salinity conditions (0 and 30 mM NaCl). Treatments T1 and T2 corresponded to the 100:0 formulation, T3 and T4 to 75:25, T5 and T6 to 0:100, and T7 and T8 to 25:75, with the second treatment of each pair receiving intermittent saline irrigation. An additional treatment (T9), consisting of the 50:50 formulation without added NaCl, was included as a control. The percentages represented the nominal relative contributions of ammonium and nitrate nitrogen rather than independent levels of total N concentration. The control was evaluated separately from the factorial analysis.

Each treatment comprised ten individually potted plants, resulting in 90 experimental units distributed over an area of approximately 48 m^2^. The rows were oriented north–south, with distances of 0.3 m between plants and 0.8 m between rows. Treatment positions were planned using Microsoft Excel to distribute the nine treatments across different rows and sections of the greenhouse, avoiding their concentration in specific areas. However, treatment allocation was not formally randomized. Each pot was labeled according to its assigned treatment and remained in the same position throughout the experimental period. Anti-aphid mesh was placed beneath the pots to prevent direct contact with the ground.

The same 90 potted plants, treatment regimes, experimental location, and cultivation cycle were used in the previously published study by Espinoza-Orozco et al. [17], which focused on vegetative growth, flowering, yield, and physical fruit-quality traits. The present manuscript reports complementary measurements of leaf gas exchange, stomatal micromorphology, and fruit biochemical composition obtained from this experimental system. These variables were evaluated separately from the previously published agronomic responses, with the biological sampling procedures described in the corresponding methodological sections.

### 4.4. Application of Treatments and Nutrient Solution Formulation

Treatment application began on 1 April 2024, and continued throughout the experimental cycle. The irrigation water had an electrical conductivity (EC) of 0.51 dS m^−1^ and a pH of 6.95, with concentrations of 4.40 meq L^−1^ Ca, 0.42 meq L^−1^ Mg, 0.27 meq L^−1^ Na, 4.09 meq L^−1^ HCO_3_^−^, 0.49 meq L^−1^ Cl^−^, and 0.28 meq L^−1^ SO_4_^2−^. Phosphoric acid (H_3_PO_4_) was added to reduce residual bicarbonate alkalinity to approximately 1.0 meq L^−1^.

The nutrient solutions were prepared using commercial fertilizers, including K_2_SO_4_, Mg(NO_3_)_2_·6H_2_O, NH_4_H_2_PO_4_, KH_2_PO_4_, KNO_3_, Ca(NO_3_)_2_·4H_2_O, CaSO_4_·2H_2_O, MgSO_4_·7H_2_O, and (NH_4_)_2_SO_4_. A micronutrient mixture containing Fe (7.5%), Mn (3.7%), B (0.4%), Zn (0.6%), Cu (0.3%), and Mo (0.2%) was also incorporated. The formulations were developed considering blueberry nutritional requirements and the findings of Frías-Ortega et al. [3]. Fertilizer application rates are presented in Table 4.

The calculated fertilizer-derived total nitrogen concentrations ranged from 79.8 to 85.1 mg L^−1^ among the eight factorial treatments, compared with 71.2 mg L^−1^ in the additional control (Table 5). Although the formulations were designed to provide relatively comparable mineral nutrition, differences in K, Ca, and S concentrations occurred because several fertilizer salts supplied more than one nutrient. Consequently, the treatments represented complete nutrient formulations rather than isolated differences in nitrogen form. The calculated mineral concentrations presented in Table 5 exclude contributions from irrigation water and phosphoric acid.

The pH of the prepared nutrient solutions was maintained between 5.5 and 6.5. The recorded EC values were 1.375, 1.192, 1.117, 0.848, and 1.171 dS m^−1^ for T1–T2, T3–T4, T5–T6, T7–T8, and the control, respectively. These measurements corresponded to the nutrient solutions used for routine fertigation and not to the separate saline irrigation solution.

Plants were irrigated daily, generally receiving approximately 1.0 L plant^−1^ day^−1^, with applied volumes ranging from 0.6 to 1.0 L depending on irrigation requirements. Drainage volume was recorded to calculate the drainage fraction, which ranged from 6% to 20%. However, the EC and pH of the drainage water and growing substrate were not monitored throughout the experiment.

For saline treatments (T2, T4, T6, and T8), the corresponding nutrient solution was replaced once per week with irrigation water containing 30 mM added NaCl, without fertilizers. On those days, the non-saline treatments (T1, T3, T5, T7, and T9) continued receiving their regular nutrient solutions. All treatments received their corresponding nutrient formulations on the remaining days. Therefore, saline treatments differed from non-saline treatments in both intermittent NaCl exposure and nutrient-solution application frequency. The 30 mM NaCl concentration refers to the applied weekly irrigation solution rather than a measured or constant root-zone salinity concentration.

### 4.5. Physiological Parameters

Leaf gas exchange measurements were performed 151 days after treatment initiation, during the fruit-filling stage, using a portable photosynthesis system (LI-6400, LI-COR Biosciences, Lincoln, NE (New England), USA (United States of America). Three plants exhibiting visually uniform growth and development were selected from each treatment. One recently matured, fully expanded leaf was evaluated per plant, resulting in three biological replicates per treatment (n = 3).

Measurements were conducted between 11:00 and 13:00 h using a 6 cm^2^ leaf chamber, with the leaf blade fully covering the measurement area. The reference CO_2_ concentration was maintained at 400 μmol mol^−1^, and the photosynthetic photon flux density (PPFD) was set at 1500 μmol m^−2^ s^−1^. Leaf temperature was not actively controlled during measurements.

Before initiating measurements, the reference and sample infrared gas analyzers were matched using the instrument’s “Match” function. After positioning each leaf inside the chamber, gas-exchange parameters were monitored until the instrument’s “Stable” indicator reached a minimum value of 2/3. Additionally, net photosynthetic rate and stomatal conductance were visually monitored, and readings were recorded once these parameters exhibited relatively stable values.

The physiological variables recorded were net photosynthetic rate (A, μmol CO_2_ m^−2^ s^−1^), stomatal conductance (g_s_, mmol H_2_O m^−2^ s^−1^), intercellular CO_2_ concentration (C_i_, μmol mol^−1^), and transpiration rate (E, mmol H_2_O m^−2^ s^−1^).

### 4.6. Micromorphological Parameters

**Figure 4:**
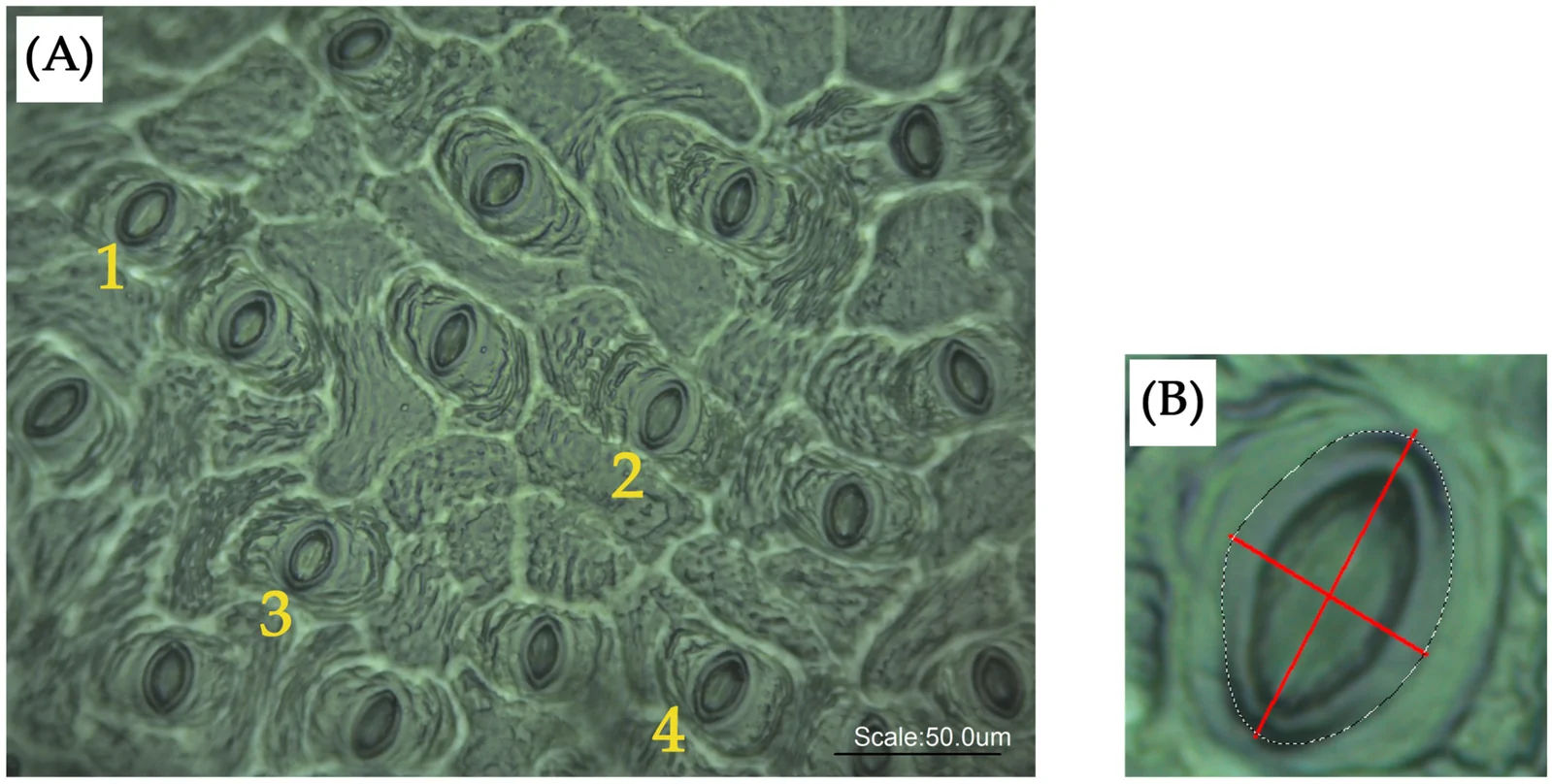
Representative photomicrographs of stomata in the abaxial leaf epidermis of blueberry (*Vaccinium corymbosum* L. cv. ‘Biloxi’). (**A**) Microscopic field showing four selected stomata (1–4) used for morphometric measurements. Scale bar = 50 µm. (**B**) Enlarged view of stomata 2 from panel (**A**), illustrating the measurement procedure. The red lines represent stomatal length (*SL*) and width (*SW*), whereas the dotted outline indicates the external boundary of the paired guard cells used to determine stomatal area (*SA*) and perimeter (*SP*). Measurements correspond to the entire stomatal structure rather than the central stomatal pore.

Stomatal micromorphology was evaluated at the end of the production cycle using mature leaves that had developed after treatment initiation. Four independent plants were evaluated per treatment, with one leaf sampled from each plant, corresponding to the sixth leaf counted from the shoot apex. Sampling was conducted between 10:00 and 15:00 h. One epidermal impression was obtained from the abaxial surface of each selected leaf using the clear nail polish technique described by Pathoumthong et al. [21]. After drying for approximately 3–5 min, the epidermal impression was transferred onto a glass microscope slide using transparent adhesive tape. One micrograph was obtained from each leaf, resulting in four micrographs per treatment and 36 micrographs across the nine treatments. Microscopic fields were selected without following a predetermined spatial pattern, ensuring that the stomata selected for morphometric analysis were fully visible. Micrographs were captured at 40× magnification using a digital binocular microscope (VE-BC3 PLUS, Velab Co., Pharr, TX, USA) equipped with a digital camera (ISH500, Tucsen Photonics Co., Ltd., Fuzhou, Fujian, China). Image acquisition was performed using Tcapture software (version 4.3.0.605). Each microscopic field covered an area of 73,500.4 µm^2^ (0.0735004 mm^2^). The numbers of stomata (S) and non-stomatal epidermal cells (E) were counted to calculate the stomatal index (SI, %) using Equation (1) and stomatal density (SD, stomata mm^−2^) using Equation (2). A representative microscopic field used for these evaluations is presented in Figure 4A.

(1) $$\mathrm{SI}=\frac{S}{S+E}\times 100$$

(2) $$\mathrm{SD}=\frac{S}{A}$$

where:

*SI* = stomatal index

*SD* = stomatal density

*S* = number of stomata

*E* = number of non-stomatal epidermal cells

*A* = sampled area

For stomatal morphometric characterization, four complete stomata were measured in each micrograph, resulting in 16 stomata per treatment and 144 stomata across the experiment. Photomicrographs obtained from epidermal impressions were analyzed using Adobe Photoshop^®^ (version 25.11.0), following image-processing approaches previously described for the morphometric characterization of plant structures [22]. Similarly, Zhu et al. [36] described manual image-based measurements for the quantification of stomatal characteristics. Each image was first calibrated using the 50-µm scale bar included in the micrograph to ensure accurate conversion from pixels to micrometers. Stomatal length (SL) and width (SW) were measured across the entire stomatal structure, including both guard cells. Subsequently, the external boundary of the paired guard cells was manually delineated using the elliptical or polygonal selection tool, according to stomatal morphology. Photoshop was then used to calculate stomatal area (SA, µm^2^) and perimeter (SP, µm) from the selected outlines. The measurement procedure is illustrated in Figure 4B, which shows an enlarged view of stoma 2 from Figure 4A. These morphometric measurements represent the entire stomatal structure rather than exclusively the central stomatal pore. Individual plants constituted the biological replicates (n = 4 per treatment), whereas the four stomata measured within each micrograph represented subsamples.

### 4.7. Biochemical Parameters

Blueberry fruits were harvested at commercial maturity, determined visually by the development of a uniform blue coloration over the entire fruit surface. Following each harvest, fruits were frozen at −18 °C and maintained in frozen storage until preparation for lyophilization. At this stage, fruits collected from different harvest dates and all ten pots within each treatment were pooled to obtain a single composite sample per treatment.

The pooled fruits were crushed while still frozen to maintain their firmness and facilitate sample processing. Immediately after crushing, the samples were transferred to a freezer at −30 °C, where they remained for five days before lyophilization. Subsequently, the frozen samples were freeze-dried for 120 h using a laboratory-scale freeze dryer. A single instrument reading during the process indicated a vacuum pressure of 61 Pa, a sample temperature of −44.9 °C, and a cold-trap temperature of −48.2 °C. Immediately after lyophilization, the dried samples were ground into a fine powder and stored in airtight bags at room temperature, protected from light, for approximately five months before biochemical analysis.

For each biochemical assay, three separate subsamples (0.200 g each) were independently weighed from the lyophilized and homogenized composite fruit sample corresponding to each treatment. Each subsample was placed in a separate tube and independently subjected to the complete extraction, centrifugation, and spectrophotometric determination procedure. Thus, three independent analytical preparations were performed per treatment for each biochemical variable. Because fruits from the ten potted plants within each treatment had been pooled before processing, these determinations represented analytical replicates rather than independent biological replicates. Total sugars (TS) were determined using the phenol–sulfuric acid colorimetric method [20,34]. Briefly, each 0.200-g subsample of lyophilized blueberry fruit powder was mixed with 10 mL of distilled water, homogenized using a vortex mixer, and centrifuged at 6000 rpm for 10 min at room temperature. A 500-µL aliquot of the undiluted supernatant from each independently prepared extract was mixed with 500 µL of 5% phenol solution, followed by the addition of 1000 µL of concentrated sulfuric acid (96–98%). The reaction mixture was incubated in a water bath at 50 °C for 5 min and subsequently cooled to room temperature. Absorbance was measured at 480 nm using a spectrophotometer.

Sucrose standard solutions ranging from 0 to 1.0 mg mL^−1^ were subjected to the same analytical procedure. Absorbance values were corrected using the reagent blank, and sucrose-equivalent concentrations were calculated using the calibration equation y = 1.3166x − 0.0182 (R^2^ = 0.9944). Results were expressed as milligrams of sucrose equivalents per gram of dry matter (mg SE g^−1^ DM). Ascorbic acid (AA) was determined spectrophotometrically using the 2,6-dichlorophenolindophenol (DCPIP) colorimetric method, adapted from Manríquez-Torres et al. [35], with modifications. For each treatment, three separate subsamples (0.200 g each) of lyophilized blueberry fruit powder were weighed from a single composite fruit sample. Each subsample was independently homogenized with 10 mL of 0.4% oxalic acid solution using a vortex mixer and subsequently centrifuged at 10,000× *g* for 10 min at 4 °C. The resulting supernatant from each independently prepared extract was used directly for analysis without additional purification.

The sodium acetate–acetic acid buffer was prepared using 300 g of sodium acetate, 700 mL of distilled water, and 1000 mL of glacial acetic acid. The DCPIP solution was prepared by dissolving 12 mg of 2,6-dichlorophenolindophenol in 1 L of distilled water.

For the colorimetric reaction, 0.25 mL of each independently prepared supernatant was mixed with 0.25 mL of sodium acetate buffer and maintained in the dark for 5 min. Subsequently, 2.0 mL of DCPIP solution (12 mg L^−1^) was added, and the reaction mixture was allowed to stand for 15 min before absorbance was measured at 520 nm using a spectrophotometer.

A reagent blank was used for background correction. Ascorbic acid standards ranging from 0 to 50 mg L^−1^ were processed under the same reaction conditions. Concentrations were calculated using the calibration equation y = 0.689 − 0.0104x (R^2^ = 0.9921), where y represents absorbance and x represents ascorbic acid concentration (mg L^−1^). Results were expressed as milligrams of ascorbic acid equivalents per gram of dry matter (mg AA g^−1^ DM). The three independent analytical preparations represented analytical replicates from a single composite sample rather than independent biological replicates.

### 4.8. Statistical Evaluation

Physiological and stomatal micromorphological data were analyzed using a two-way factorial analysis of variance (ANOVA), considering nitrogen formulation (four levels), NaCl salinity (two levels), and their interaction as fixed effects. The factorial analysis included treatments T1–T8, whereas the additional 50:50 NH_4_^+^–N_3_^−^–N control (T9) was evaluated separately. Individual potted plants constituted the experimental units, with three biological replicates per treatment for gas-exchange measurements and four for stomatal micromorphology. Individual stomatal measurements were treated as subsamples and averaged at the plant level before analysis. Comparisons among nitrogen formulations within each salinity condition were performed using Tukey-adjusted tests, while salinity contrasts within each formulation were evaluated using Holm-adjusted comparisons. Differences between each factorial treatment and the additional control were assessed using two-sided Dunnett tests from a separate one-way ANOVA. For physiological variables, residual normality and homogeneity of variances were evaluated using the Shapiro–Wilk and Levene tests, respectively. Statistical significance was established at *p* < 0.05. Fruit biochemical data were analyzed descriptively and expressed as means ± analytical standard deviations from three independently processed subsamples of a single composite fruit sample per treatment. No inferential statistical tests were performed on biochemical variables because independent biological replication was unavailable. Statistical analyses and graphical representations were performed using R version 4.5.2 [37].

## 5. Conclusions

The physiological response of ‘Biloxi’ blueberry to intermittent NaCl irrigation depended on the nutrient formulation. Significant formulation × salinity interactions were detected for *A*, *g_s_*, *C_i_*, and *E*. Salinity reduced net photosynthesis under the 100:0, 0:100, and 25:75 NH_4_^+^–NO_3_^−^–N formulations, whereas the 75:25 formulation exhibited an opposite response.

Nitrogen formulation significantly affected stomatal index and density, with the highest numerical values observed under the 25:75 formulation without added NaCl. However, salinity and its interaction with formulation did not significantly affect the evaluated micromorphological traits, and no significant treatment effects were detected for guard-cell dimensions.

Fruit total sugars and ascorbic acid showed descriptive variation among treatments. Because these determinations were obtained from a single composite biological sample per treatment, their numerical differences cannot establish treatment-related biochemical effects.

Overall, the results indicate that the physiological performance of ‘Biloxi’ blueberry depends on the specific combination of nutrient formulation and saline irrigation. Nevertheless, differences in accompanying mineral nutrients, the intermittent application of NaCl, and the absence of root-zone salinity monitoring limit the attribution of these responses exclusively to nitrogen form or salinity. Additional experiments are required before establishing specific fertilization recommendations for blueberry production under saline conditions.

## Figures and Tables

**Table 1:** Gas-exchange responses of blueberry plants to nitrogen formulations and NaCl salinity, including factorial ANOVA.

Treatment	NH_4_^+^–N:NO_3_^−^–N	NaCl (mM)	*A*	*g_s_*	*C_i_*	*E*
T1	100:0	0	3.670 ± 0.141 b	0.0630 ± 0.0261 a	260.7 ± 47.6 a	1.997 ± 0.536 a
T2	100:0	30	1.397 ± 0.330 c^*†^	0.0646 ± 0.0306 a	339.3 ± 11.2 a^*†^	1.937 ± 0.692 ab
T3	75:25	0	2.733 ± 0.245 c^†^	0.0469 ± 0.0089 a	275.0 ± 26.0 a	1.707 ± 0.155 a
T4	75:25	30	4.500 ± 0.428 a^*^	0.0717 ± 0.0265 a	261.7 ± 36.2 b	2.607 ± 0.935 a
T5	0:100	0	5.260 ± 0.773 a^†^	0.0847 ± 0.0085 a	272.7 ± 9.8 a	2.797 ± 0.095 a
T6	0:100	30	2.667 ± 0.295 b^*†^	0.0444 ± 0.0125 a	273.3 ± 17.0 ab	1.507 ± 0.395 ab^*^
T7	25:75	0	4.723 ± 0.035 a^†^	0.0713 ± 0.0240 a	251.7 ± 40.5 a	2.590 ± 0.785 a
T8	25:75	30	1.717 ± 0.142 c^*†^	0.0317 ± 0.0195 a	330.0 ± 16.8 a^*^	1.028 ± 0.495 b^*^
T9 (Ctrl)	50:50	0	3.730 ± 0.606	0.0594 ± 0.0305	251.7 ± 69.6	2.057 ± 0.774
FACTORIAL ANOVA (T1–T8) · F statistic; *p* value			***A***	***g_s_***	***C_i_***	***E***
Formulation (F) (df = 3; error df = 16)			F = 16.77; *p* <0.0001	F = 0.48; *p* = 0.7014	F = 1.59; *p* = 0.2306	F = 0.50; *p* = 0.6908
Salinity (S) (df = 1; error df = 16)			F = 103.75; *p* <0.0001	F = 2.40; *p* = 0.1405	F = 9.34; *p* = 0.0075	F = 4.51; *p* = 0.0497
F × S (df = 3; error df = 16)			F = 54.65; *p* <0.0001	F = 3.45; *p* = 0.0417	F = 4.36; *p* = 0.0200	F = 5.80; *p* = 0.0070

Values are mean ± SD (n = 3 independent potted plants per treatment). Lowercase letters compare the four nitrogen formulations within each salinity level and variable (Tukey, *p* < 0.05); letters are interpreted separately at 0 and 30 mM NaCl. A superscript * at 30 mM denotes a significant salinity contrast within the corresponding formulation (Holm-adjusted *p* < 0.05). A superscript † indicates a significant contrast with the single 50:50 control (Dunnett-adjusted *p* < 0.05). The control is not included in the 4 × 2 ANOVA and therefore has no Tukey letter. Net photosynthetic rate (*A*, µmol CO_2_ m^−2^ s^−1^), stomatal conductance (*g_s_*, mmol H_2_O m^−2^ s^−1^), intercellular CO_2_ concentration (*C_i_*, µmol CO_2_ mol^−1^) and transpiration rate (*E*, mmol H_2_O m^−2^ s^−1^).

**Table 2:** Leaf stomatal micromorphological traits of blueberry cv. ‘Biloxi’ under four nitrogen formulations and two NaCl levels, including factorial ANOVA.

Treatment	NH_4_^+^–N:NO_3_^−^–N	NaCl (mM)	*SI* (%)	*SD* (Stomata mm^−2^)	*SL* (µm)	*SW* (µm)	*SA* (µm^2^)	*SP* (µm)
T1	100:0	0	25.09 ± 4.47 b	190.48 ± 40.05 b	23.50 ± 2.84 a	14.88 ± 1.45 a	260.96 ± 40.31 a	63.01 ± 5.49 a
T2	100:0	30	25.81 ± 2.76 b	207.48 ± 23.24 a	22.44 ± 0.31 a	14.81 ± 0.55 a	248.86 ± 9.90 a	61.31 ± 1.37 a
T3	75:25	0	24.46 ± 3.71 b^†^	200.68 ± 39.08 b	21.94 ± 1.14 a	14.25 ± 0.54 a	244.51 ± 19.20 a	60.60 ± 2.29 a
T4	75:25	30	29.64 ± 1.70 ab	234.69 ± 20.41 a	21.38 ± 2.38 a	13.94 ± 1.03 a	231.27 ± 34.57 a	58.73 ± 4.83 a
T5	0:100	0	30.06 ± 1.65 b	251.70 ± 17.56 ab	23.62 ± 0.52 a	14.88 ± 0.75 a	270.29 ± 19.26 a	63.70 ± 1.82 a
T6	0:100	30	31.97 ± 4.62 ab	265.30 ± 39.28 a	22.81 ± 0.31 a	15.00 ± 0.94 a	255.97 ± 21.51 a	62.20 ± 2.12 a
T7	25:75	0	38.19 ± 5.76 a	289.11 ± 32.15 a	22.25 ± 1.06 a	14.38 ± 0.43 a	235.50 ± 12.52 a	60.02 ± 1.81 a
T8	25:75	30	33.11 ± 2.47 a	244.90 ± 33.33 a	23.75 ± 0.46 a	15.19 ± 0.69 a	273.46 ± 19.67 a	64.55 ± 2.11 a
T9 (Ctrl)	50:50	0	32.38 ± 4.62	244.90 ± 22.22	23.12 ± 2.42	15.00 ± 1.04	253.33 ± 42.08	62.01 ± 5.15
FACTORIAL ANOVA (T1–T8): F statistic (*p* value); residual df = 24								
Formulation (F) df = 3			F = 12.33; *p* = 4.44 × 10^−5^	F = 8.37; *p* = 0.00055	F = 1.93; *p* = 0.15231	F = 1.62; *p* = 0.21007	F = 1.54; *p* = 0.23037	F = 1.75; *p* = 0.18288
Salinity (S) df = 1			F = 0.28; *p* = 0.60450	F = 0.21; *p* = 0.65392	F = 0.21; *p* = 0.65183	F = 0.22; *p* = 0.64629	F = 0.00; *p* = 0.96077	F = 0.02; *p* = 0.90291
F × S df = 3			F = 2.72; *p* = 0.06695	F = 2.30; *p* = 0.10313	F = 1.31; *p* = 0.29418	F = 0.64; *p* = 0.59938	F = 2.25; *p* = 0.10837	F = 2.04; *p* = 0.13514
Error df = 24			MSE = 13.445	MSE = 1010.370	MSE = 2.105	MSE = 0.732	MSE = 582.992	MSE = 9.504

Lowercase letters compare the four nitrogen formulations within each salinity level and variable (Tukey, *p* < 0.05); letters are interpreted separately at 0 and 30 mM NaCl. A superscript † indicates a significant contrast with the single 50:50 control (Dunnett-adjusted *p* < 0.05). The control was not included in the 4 × 2 factorial ANOVA and therefore has no Tukey letter. Stomatal index (*SI*, percent %), stomatal density (*SD*, stomata mm^−2^), stomatal length (*SL*, µm), stomatal width (*SW*, µm), stomatal area (*SA*, µm^2^), and stomatal perimeter (*SP*, µm).

**Table 3:** Descriptive total sugar and ascorbic acid contents of blueberry fruit under nitrogen formulations and NaCl salinity.

Treatment	NH_4_^+^–N:NO_3_^−^–N	NaCl (mM)	Total Sugars (TS) (mg SE g^−1^ DM)	Ascorbic Acid (AA) (mg AA g^−1^ DM)
T1	100:0	0	40.28 ± 0.64	2.402 ± 0.048
T2	100:0	30	26.49 ± 0.12	2.261 ± 0.025
T3	75:25	0	30.27 ± 0.22	2.522 ± 0.015
T4	75:25	30	35.45 ± 0.41	2.527 ± 0.019
T5	0:100	0	33.66 ± 0.04	2.266 ± 0.003
T6	0:100	30	34.49 ± 0.40	2.459 ± 0.047
T7	25:75	0	32.62 ± 0.39	2.514 ± 0.058
T8	25:75	30	31.55 ± 0.69	2.515 ± 0.037
T9 (Ctrl)	50:50	0	42.35 ± 1.25	2.504 ± 0.079

Values are means ± analytical SD from three independently prepared analytical subsamples per treatment (n = 3 analytical preparations). SE, sucrose equivalents; AA, ascorbic acid; DM, dry matter.

**Table 4:** Fertilizers used in the formulation of nutrient solutions for experimental treatments.

Source	T1–T2	T3–T4	T5–T6	T7–T8	Control
	g L^−1^				
K_2_SO_4_	0.17	0.17	0.04	0.11	0.13
Mg(NO_3_)_2_·6H_2_O			0.22	0.22	
NH_4_H_2_PO_4_	0.08	0.08			
KH_2_PO_4_			0.09	0.09	0.09
KNO_3_			0.19	0.03	
Ca(NO_3_)_2_·4H_2_O		0.14	0.26	0.26	0.26
CaSO_4_·2H_2_O	0.22	0.1	0.06		
MgSO_4_·7H_2_O	0.19	0.19			0.19
(NH_4_)_2_·SO_4_	0.35	0.25		0.09	0.16
Micronutrient Complex	0.02	0.02	0.02	0.02	0.02

**Table 5:** Calculated mineral nutrient concentrations supplied by the fertilizers used in the experimental nutrient solutions.

Formulation	NH_4_^+^–N	NO_3_^−^–N	N Total	P	K	Ca	Mg	S
	mg L^−1^							
T1–T2	82	0	82	19.8	70.7	48	19.7	116.5
T3–T4	60.4	19.4	79.8	19.8	70.7	47.9	19.7	88.3
T5–T6	0	85	85	19.7	113	61.1	19.7	19.3
T7–T8	19.5	65.6	85.1	19.7	80.5	47.9	19.7	26.8
Control	34	37.2	71.2	19.7	80.3	47.9	19.7	57.2

All concentrations are expressed in mg L^−1^ and were calculated based on the declared nutrient composition of the commercial fertilizers. The values represent fertilizer-derived nutrient contributions only and exclude the mineral elements naturally present in the irrigation water. The nominal NH_4_^+^-NO_3_^−^-N ratios were 100:0, 75:25, 0:100, 25:75, and 50:50 for T1–T2, T3–T4, T5–T6, T7–T8, and the control, respectively.

## Data Availability

The data presented in this study are available in article.
